# [Retracted] Paclitaxel inhibits breast cancer metastasis via suppression of Aurora kinase‑mediated cofilin‑1 activity

**DOI:** 10.3892/etm.2024.12598

**Published:** 2024-06-03

**Authors:** Yue Zhang, Yaoyi Wang, Jun Xue

Exp Ther Med 15:1269–1276, 2018; DOI: 10.3892/etm.2017.5588

Following the publication of this paper, it was drawn to the Editor’s attention by a concerned reader that certain of the migration assay data shown in Fig. 2B on p. 1272 were strikingly similar to data appearing in different form in another article written by different authors at different research institutes that had already been published in the journal *Molecular Cancer Research* prior to the submission of this paper to *Experimental and Therapeutic Medicine*. In view of the fact that the abovementioned data had already apparently been published previously, the Editor of *Experimental and Therapeutic Medicine* has decided that this paper should be retracted from the Journal. The authors were asked for an explanation to account for these concerns, but the Editorial Office did not receive a satisfactory reply. The Editor apologizes to the readership for any inconvenience caused.

